# The effect of diabetes on short-term outcomes following epiretinal membrane surgery

**DOI:** 10.1007/s10792-024-03373-6

**Published:** 2024-12-05

**Authors:** Idan Hecht, Minna Karesvuo, Piotr Kanclerz, Sohee Jeon, Petteri Karesvuo, Raimo Tuuminen

**Affiliations:** 1 Helsinki Retina Research Group, University of Helsinki, Helsinki, Finland; 2 Sackler School of Medicine, Tel Aviv University, Tel Aviv, Israel; 3 Department of Ophthalmology, Shamir Medical Center, Tel Aviv, Israel; 4 Department of Oral and Maxillofacial Diseases, Kuopio University Hospital, Kuopio, Finland; 5 Hygeia Clinic, Gdańsk, Poland; 6 Vitreoretinal Service, Keye Eye Center, 326 Teheran-Ro, Gangnam-Gu, Seoul, South Korea; 7 Department of Ophthalmology, Helsinki University Hospital, Helsinki, Finland; 8 Department of Ophthalmology, Kymenlaakso Central Hospital, Kotkantie 41, 48210 Kotka, Finland

**Keywords:** Diabetes, Epiretinal membrane surgery, ERM peeling, Glycemic control, Insulin medication

## Abstract

**Purpose:**

This study aimed to examine the association of diabetes with anatomical and functional outcomes of epiretinal membrane surgery.

**Methods:**

Consecutive patients who underwent epiretinal membrane surgery between 2017–2021 at Helsinki University Hospital, Finland. Here we examined the association of diabetes, glycemic control, and requirement for insulin medication with surgical outcomes at 1-month.

**Results:**

Included were 214 eyes of 214 patients, with a mean age of 71.2 ± 8.2 years. Among patients with diabetes (n = 45), neither significant differences were observed in anatomical outcomes (− 47.8 ± 72.7 μm vs. − 38.3 ± 103 μm for foveal thickness, *p* = 0.566 and − 41.6 ± 61.8 μm vs. − 41.7 ± 85.7 μm for central subfield macular thickness, *p* = 0.996) nor in best-corrected visual acuity (BCVA) gain (0.06 ± 0.22 vs 0.12 ± 0.30 LogMAR units, *p* = 0.214) compared to those without diabetes. In a multivariate analysis adjusted for age, gender, the existence of preoperative macular cysts, and topical nonsteroidal anti-inflammatory drugs (NSAIDs) use, the results remained consistent. The last preoperative HbA1c levels did not correlate with changes in foveal (Pearson’s r = 0.218, *p* = 0.264) or central subfield macular thickness (r = 0.365, *p* = 0.056), or with BCVA gain (r = -0.177, *p* = 0.386). Insulin therapy for diabetes did not associate with the outcomes (*p* > 0.05 for anatomical and functional comparisons).

**Conclusions:**

In a cohort of patients who underwent epiretinal membrane surgery, neither the presence of diabetes, nor glycemic control and the use of insulin medication associated with the outcomes.

**Supplementary Information:**

The online version contains supplementary material available at 10.1007/s10792-024-03373-6.

## Introduction

Epiretinal membrane (ERM) formation has been linked with various inflammatory mechanisms including both cellular inflammatory components and inflammatory mediators such as cytokines and growth factors [1].

Diabetes mellitus is also known to increase inflammatory pathways associated with retinal pathology and can be associated with the formation of epiretinal membranes (ERMs) [2–4]. Diabetes is also the cause of diabetic retinopathy (DR), which is associated with worse outcomes following ERM surgery [5, 6]. Previous studies have shown that following surgery for ERM, patients with diabetic retinopathy had a higher likelihood of developing macular atrophy or scarring, a higher percentage of inner segment/outer segment junction disruption, slower anatomical improvement postoperatively, and higher ERM recurrence [5, 6]. However, it is unclear whether diabetes is associated with worse anatomical or functional surgical outcomes, as current evidence is conflicting [7].

Here we aimed to examine the possible influences of diabetes, including glycemic control, and systemic diabetes treatment, on ERM surgery outcome parameters.

## Methods

### Study design

The study was approved by the Hospital District of Helsinki University Hospital, Helsinki, Finland (§42 / 2022; HUS/148/2022), and it adhered to the tenets of the Declaration of Helsinki. This study was a retrospective registry-based cohort study of consecutive ERM surgeries performed between January 2017 and October 2021 at the Department of Ophthalmology, Helsinki University Hospital. The source of the information was electronic patient medical records of Helsinki University Hospital and software for optical coherence tomography (OCT) scans.

Prior to surgery and postoperatively, all patients underwent a complete ophthalmological examination including assessment of best-corrected visual acuity (BCVA), anterior segment and fundus with slit-lamp (biomicroscope), as well as retinal OCT.

The main outcome was defined as gain in BCVA at 1-month. Anatomical changes were defined as secondary outcomes and included changes in foveal, maximal, and central subfield macular thickness (CSMT; here defined as mean thickness in the central 1000-$\mu$ m diameter area). All patients were followed for one month. According to the Helsinki University Hospital guidelines, patients routinely continued their long-term follow-up in the community-based sector.

### Clinical evaluation and postoperative care

Patients were diagnosed, operated, and followed according to the Helsinki University Hospital guidelines. Concomitant ILM peeling was performed according to operating surgeons’ preference. The diagnosis and preoperative assessment were performed by a vitreoretinal surgeon. OCT analysis was done in all cases pre- and postoperatively (Heidelberg Engineering GmbH, Heidelberg, Germany). International classification of diseases (ICD)-coding was used to specify diagnosis; ICD-10 H35.38# for macular preretinal fibrosis. Medical Retina specialist (P.K.) reviewed the OCT scans and the automated retinal thickness analyses produced by the Heidelberg software.

Postoperative care included routine use of levofloxacin 5 mg/ml eye drops (Oftaquix^®^, Santen Pharmaceutical Co. Ltd., Osaka, Japan) four times a day for 2 weeks and either combined dexamethasone phosphate 1 mg/ml and chloramphenicol 2 mg/ml eye drops (Oftan Dexa-Chlora^®^, Santen Pharmaceutical Co. Ltd.) or prednisolone acetate (Pred Forte^®^, 10 mg/ml, AbbVie Inc., North Chicago, IL) four times a day for one month, depending on the surgeon’s preference. Intraoperative intravitreal triamcinolone acetonide suspension (40 mg/ml, Triesence^®^, Novartis, Basel, Switzerland) at the end of surgery and postoperative nepafenac (3 mg/ml, Nevanac^®^, Novartis) once daily, Diclofenac (1 mg/ml, Voltaren Ophtha^®^, Théa Laboratoires, Clermont Ferrand, France) t.i.d., or Bromfenac (0.9 mg/ml, Yellox^®^, Bausch & Lomb, Laval, Canada) b.i.d. were given for one to three months to some of the patients depending on the surgeon’s preference.

### Inclusion and exclusion criteria

ERM surgeries with at least one-month of follow-up data were included in the study. Exclusion criteria were concomitant phacoemulsification surgery, prior vitrectomy for any reason, prior or present uveitis, macular hole, retinal vein occlusion, vitreomacular traction, proliferative diabetic retinopathy, anti-VEGF treatment for any reason, and missing data for the diabetic status.

### Sample size calculation

For sample calculations the primary outcome measure of BCVA gain between the groups was used. Important differences in BCVA are usually considered to range between ten to fifteen ETDRS letters (two to three ETDRS lines, equivalent to 0.2–0.3 LogMAR) [6, 7]. Here, two ETDRS lines (0.2 LogMAR) were considered as a clinically important difference. Standard deviation of visual gain was based on previous publications and set at 0.3 LogMAR. A sample size of 36 patients for each group (total of 72) was required to detect a significant difference between the groups with a significance level of 0.05 and a power of 80% using an independent model.

### Statistics

To avoid biases arising from between-eye correlation, a single eye of each patient was included in the study. Unless otherwise specified, data is presented as mean ± standard deviation (SD). BCVA decimal values were converted to logarithm of the minimum angle of resolution (LogMAR) for statistical purposes. Clinical parameter distributions were tested for normality by the Shapiro–Wilk test. Intraclass correlation coefficient (ICC) was calculated, and it indicated good reliability (ICC between 0.75 and 0.9) for primary (gain in BCVA at 1-month) and secondary (changes in foveal, maximal, and central subfield macular thickness at -month) outcome measures. In the analysis, we first examined the baseline parameters of patients with and without diabetes. For categorical variables the χ2 test was used, or the Fisher’s exact test as appropriate. Independent T-test was conducted for continuous variables with a normal distribution and the Mann–Whitney U-test for variables with a non-normal distribution. Next, the anatomical and functional changes of the entire group were analyzed. The McNemar test was used to analyze paired nominal data. A Paired T-test and the Wilcoxon test were used for continuous variables with normal and non-normal distribution as appropriate. Subsequently, the main functional and anatomical outcomes of patients with and without diabetes were examined using both univariate and multivariate analyses. Multivariate analyses were done using binary logistic regression for an effect size above the median, controlling for possible confounders. Next, we focused on the group with diabetes, and examined all possible effects of the type of treatment, glycemic control, and associated systemic factors on various outcomes. This was done using bivariate correlation tests for continuous variables (Pearson correlation for normally distributing variables and Spearman correlation for non-normally distributing variables).

Statistical analysis was performed using IBM SPSS Statistics 27 (IBM Corp. Armonk, NY). *p* < 0.05 on a two-sided test was considered statistically significant.

## Results

The mean age of the 214 patients (214 eyes) included was 71.2 ± 8.2 years. Male-to-female gender distribution was 51% to 49%. Nine percent (N = 20) were current smokers. Baseline characteristics of the patients with and without diabetes are available in Table 1.

**Table 1:** Baseline clinical variables among patients without and with diabetes

Variable	Diabetes		*P*-value
	No	Yes	
N	169	45	
Age (years)	71.2 ± 8.1	71.3 ± 8.6	.924
*Diabetes type*			
Type 1	–	7 (15.6)	
Type 2	–	37 (82.2)	
LADA	–	1 (2.2)	
Current smoker	16 (9.5)	4 (8.9)	.887
Male:female	86:83 (51:49)	24:21 (53:47)	.885
Laterality (OD)	84 (49.7)	22 (48.9)	.827
Pseudophakia	81 (47.9)	22 (48.9)	.909
Glaucoma	20 (11.8)	6 (13.3)	.784
Use of Triamcinolone	21 (12.4)	6 (13.3)	.988
23:25:27G vitrectomy*	10:111:39 (6:66:23)	3:31:10 (7:69:22)	.969
Dual blue was used	128 (75.7)	37 (82.2)	.536
Topical NSAIDs (any)	27 (16.0)	9 (20.0)	.521
*Preoperative clinical attributes*			
BCVA (LogMAR)	0.44 ± 0.33	0.45 ± 0.25	.944
Preop macular cysts	48 (28.5)	21 (46.6)	.024
Foveal thickness	457 ± 100	439 ± 92	.286
Max thickness	514 ± 107	486 ± 83	.100
CSMT	464 ± 83	442 ± 77	.105
*Medical treatment*			
ACE/AT-2	66 (39.1)	26 (57.8)	.026
Aspirin**	16 (9.5)	6 (13.3)	.456
Beta-blockers	32 (18.9)	26 (57.8)	< 0.001
BPH medications	11 (6.5)	4 (8.9)	.586
CCB	34 (20.1)	25 (55.6)	< 0.001
Diuretics	24 (14.2)	14 (31.1)	.009
Insulin	0 (0)	17 (37.8)	< 0.001
Nitrates	6 (3.6)	6 (13.3)	.012
Statins (any)	67 (39.6)	28 (62.2)	.007

Baseline clinical, surgical, and ophthalmic variables among patients with and without diabetes undergoing ERM surgery. Data is given as mean ± SD or absolute numbers (with proportions). ACE/AT-2; angiotensin-converting-enzyme inhibitors / angiotensin-2 receptor blockers, BCVA; best corrected visual acuity, BPH; benign prostatic hyperplasia, CCB; calcium channel blockers, CSMT; central subfield macular thickness (mean thickness in the central 1000-$\mu$ m diameter area), ERM; epiretinal membrane, LADA; latent autoimmune diabetes of adults, LogMAR; Logarithm of the Minimum Angle of Resolution, NSAIDs; non-steroidal anti-inflammatory drugs. * = data not available for 10 cases. **available in pharmacies also without prescription

At 1-month, the mean foveal thickness decreased from 453.2 µm to 413.2 µm (*p* < 0.001), maximal thickness decreased from 508.4 µm to 468.7 µm (*p* < 0.001), and CSMT decreased from 459.7 µm to 418.0 µm (*p* < 0.001). Concomitantly, BCVA improved from 0.442 LogMAR units to 0.335 LogMAR units (*p* < 0.001).

### Patients with and without diabetes

Of the 214 included cases, 45 had diabetes. Type 2 diabetes was predominant (37 patients), seven had type 1 diabetes and one had latent autoimmune diabetes of adults (LADA). As seen in Table 1, patient demographics, surgical, and clinical characteristics were similar between the groups at baseline. In addition to diabetes medication, the groups differed in some aspects of systemic medical therapy, most notably beta-blockers and calcium channel blockers (Table 1). Furthermore, patients with diabetes were significantly more likely to have preoperative macular cysts compared to those without diabetes (46.6% vs. 28.5% respectively, *p* = 0.024).

In the univariate analysis, no significant differences were seen in the BCVA gain among patients with diabetes (0.06 ± 0.22 LogMAR units) and those without (0.12 ± 0.30 LogMAR units, *p* = 0.214, Table 2), nor in anatomical outcomes including foveal thickness (− 47.8 ± 72.7 μm vs. − 38.3 ± 103 μm, *p* = 0.566, Table 2), maximal thickness (− 28.6 ± 69.7 μm vs. − 42.5 ± 109 μm, *p* = 0.419, Table 2) and CSMT (− 41.6 ± 61.8 μm vs. − 41.7 ± 85.7 μm, *p* = 0.996, Table 2), respectively.

**Table 2:** Functional and anatomical outcomes among patients without and with diabetes

Variable	Diabetes		*P*-value
	No	Yes	
*Main outcome*			
BCVA gain (LogMAR)	0.12 ± 0.30	0.06 ± 0.22	.214
*Secondary outcomes*			
Change in foveal thickness (µm)	− 38.3 ± 103.7	− 47.8 ± 72.7	.566
Change in max thickness (µm)	− 42.5 ± 109.4	− 28.6 ± 69.7	.419
Change in CSMT (µm)	− 41.7 ± 85.7	− 41.6 ± 61.8	.996

Main functional and anatomical outcomes in a univariate (un-adjusted) analysis among patients with and without diabetes undergoing epiretinal membrane surgery. Data is given as mean ± SD. BCVA; best-corrected visual acuity, CSMT; central subfield macular thickness (mean thickness in the central 1000-$\mu$ m diameter area), LogMAR; Logarithm of the Minimum Angle of Resolution

In the multivariate analysis adjusted for age, gender, the existence of preoperative macular cysts, and pseudophakic status, again no significant difference was seen in the BCVA gain (odds ratio [OR] for BCVA gain above the median for patients with diabetes: 0.537, 95% confidence interval (95% CI): 0.247–1.168, *p* = 0.184). When adjusted for topical NSAID use, the results remain consistent (OR: 0.656, 95% CI: 0.325–1.323, *p* = 0.239).

Despite the higher prevalence of preoperative macular cysts among patients with diabetes, no significant difference in prevalence of macular cysts was seen postoperatively among patients with (n = 19, 42.2%) and without diabetes (n = 60, 35.5%, *p* = 0.110, Supplemental Table 1).

### Sub-analysis: patients with diabetes

In the individuals with diabetes (n = 45), the possible effects of the type of treatment and glycemic control on functional and anatomical outcomes was examined. HbA1c levels were not significantly correlated with anatomical (Pearson’s r = 0.218, *p* = 0.264 for change in foveal thickness; r = 0.251, *p* = 0.198 for change in maximal thickness; and r = 0.365, *p* = 0.056 for change in CSMT) or functional outcomes (r = − 0.177, *p* = 0.386 for BCVA gain). No significant differences in anatomical and functional outcomes were noted among patients treated with insulin (n = 17) compared to those without it (n = 28), nor among smokers compared to non-smokers (*p* > 0.05 for all comparisons).

## Discussion

In this cohort of patients who underwent ERM surgery, diabetes, glycemic control or need for insulin medication, were not associated with worse outcomes, regardless of topical NSAIDs anti-inflammatory adjunct therapy.

For severe ERMs, surgery is widely considered to improve outcomes [8]. However, the influence of underlying conditions such as diabetes itself on the outcomes of ERM surgery has remained unclear [9]. In this study, the systemic aspects related to diabetes and treatment of diabetes were examined, as well as potential influences of adjunct postoperative anti-inflammatory therapy. Eyes with a concomitant diagnosis of proliferative diabetic retinopathy or with history of anti-VEGF treatment were excluded, in order to focus on the effects of diabetes in itself and not on the effects of overt complications related to the disease. The results show that the degree of improvement was comparable between patients with and without diabetes. Poor glycemic control or need for insulin treatment were not associated with worse surgical outcomes and should not result in postponing the surgery. Early surgical interventions resulted in favorable anatomical and functional improvement among diabetic patients with macular edema [10, 11]. Interestingly, concomitant resolution of outer retinal hyperreflective deposits was found after operative intervention [12]. In addition to surgery itself, co-adjuvant intravitreal steroids [13–19] and anti-VEGF agents [20–23] effectively reduced macular edema, proliferative vitreoretinopathy severity, and ameliorated disorganization of retinal inner layers both in previously vitrectomized and non-vitrectomized eyes. Furthermore, combination of OCT and metabolic biomarkers could serve as functional outcome predictors [24, 25].

Among patients with diabetes, ERMs may present unique morphological and clinical features. Macular morphology can show notable distinctions; eyes with diabetic ERMs have more macular cystoid spaces and demonstrate thicker central fovea compared to eyes with idiopathic ERMs [4]. ERMs among patients with diabetes are more likely to be characterized by focal retinal adhesion [9]. In general, greater foveal thickness values may indicate localized traction and more severe ganglion cell damage [26]. The presence of concurrent macular edema has been shown to be a poor prognostic factor for macular thickness improvement [27]. Clinically there are also notable differences, although idiopathic and diabetic ERMs demonstrate improvement after vitrectomy, in diabetic ERMs the macula appears to be more vulnerable to damage induced by the peeling procedures [28]. The peeling seems to cause more substantial harm when there is a thicker and more rigid internal limiting membrane (ILM), leading to damage in the outer retina and structural collapse of the retina. Finally, in diabetic patients, the reduction in macular volume has been linked to the contraction of Müller cells, which is induced by ILM peeling [29].

This study has several limitations. First, the follow-up time for the patients in our study was only one month. According to the Helsinki University Hospital guidelines, patients routinely continued their long-term follow-up in the community-based sector, hence long-term follow-up data was not available. Improvement in visual acuity can be observed even after 12 months following surgery [30]. However, it has been shown that early postoperative anatomical changes are predictive of long-term anatomical outcomes [31]. Restoration of the retinal anatomical structure predominantly occurred within the first 3 months of post-ERM peeling [32]. Central macular thickness decreased steadily until 6 months after epiretinal membrane (ERM) removal surgery with no further decrease between 6 and 12 months (386 ± 73 to 390 ± 73 μm) [33]. Interestingly, early follow-up was indicative of functional results as well [34]. At 3 months, 82% of the operated patients improved their metamorphopsia according to analysis of eight modified Amsler charts test [34]. Previous studies analyzed prognostic OCT biomarkers, such as ellipsoid and interdigitation zones as well as photoreceptor outer segment length [35–39]. These factors were unfortunately beyond the scope of this study, but our further studies will hopefully help to clarify the role of prognostic OCT biomarkers in ERM peeling among patients with diabetes. Furthermore, diabetic retinopathy fundus pictures were not analyzed; however, our exclusion criteria relied on patient medical records for diagnoses, and posterior segment comorbidities were further ruled out by experienced vitreoretinal surgeons who did the preoperative assessment. We used only single imaging modality (SD-OCT) for the detection and follow-up of ERM, different imaging modalities such as swept-source and enhanced depth imaging (EDI) and deep learning system could have improved the process of detecting and following the disease and surgery outcomes [40–42].

To conclude, among patients undergoing ERM surgery, diabetes, and aspects related to glycemic control such as HbA1c levels and a requirement for insulin medication were not associated with worse surgical outcomes, regardless of topical NSAID anti-inflammatory adjunct therapy.

## Supplementary Information

Below is the link to the electronic supplementary material.

Supplementary file1 (DOCX 15 KB)

## Data Availability

No datasets were generated or analysed during the current study.
