# Supplementary material for: The effect of diabetes on short-term outcomes following epiretinal membrane surgery
Source: Int Ophthalmol. 2024 Dec 5;44(1):446. doi: 10.1007/s10792-024-03373-6 (PMC11621169; doi:10.1007/s10792-024-03373-6)
Supplement: Supplementary file 1 — Supplementary file1 (DOCX 15 KB) [file 10792_2024_3373_MOESM1_ESM.docx]

**Supplemental Table 1.** Changes in functional and anatomical aspects following epiretinal membrane surgery regarding diabetes status.

| **Patients without diabetes (n=169)** | |  | |  |  |
| --- | --- | --- | --- | --- | --- |
|  | Baseline |  | Postoperatively |  | *P*-value |
| BCVA (LogMAR) | 0.44±0.33 |  | 0.32±0.26 |  | <.001 |
| Macular cysts | 48 (28.4) |  | 60 (35.5) |  | .508 |
| Foveal thickness | 457.6±100.7 |  | 419.8±84.6 |  | <.001 |
| Max thickness | 514.8±107.9 |  | 472.2±77.1 |  | <.001 |
| CSMT | 464.8±83.2 |  | 423.1±67.8 |  | <.001 |
| **Patients with diabetes (n=45)** | | | | | |
|  | Baseline |  | Postoperatively |  | *P*-value |
| BCVA (LogMAR) | 0.45±0.25 |  | 0.39±0.25 |  | .081 |
| Macular cysts | 21 (46.6) |  | 19 (42.2) |  | .135 |
| Foveal thickness | 439.8±92.6 |  | 392.0±76.7 |  | <.001 |
| Max thickness | 486.2±83.5 |  | 457.5±67.9 |  | .009 |
| CSMT | 442.4±77.3 |  | 400.8±59.2 |  | <.001 |

Change in functional and anatomical aspects following epiretinal membrane surgery among patients with and without diabetes. Data is given as mean ± SD or absolute numbers (with proportions). BCVA; best-corrected visual acuity, CSMT; central subfield macular thickness (mean thickness in the central 1000-$\mu$m diameter area), LogMAR; Logarithm of the Minimum Angle of Resolution.
